# Health Outcomes from Home Hospitalization: Multisource Predictive Modeling

**DOI:** 10.2196/21367

**Published:** 2020-10-07

**Authors:** Mireia Calvo, Rubèn González, Núria Seijas, Emili Vela, Carme Hernández, Guillem Batiste, Felip Miralles, Josep Roca, Isaac Cano, Raimon Jané

**Affiliations:** 1 Institute for Bioengineering of Catalonia (IBEC) Barcelona Institute of Science and Technology (BIST) Universitat Politècnica de Catalunya (UPC), CIBER-BBN Barcelona Spain; 2 Hospital Clínic de Barcelona Institut d’Investigacions Biomèdiques August Pi i Sunyer (IDIBAPS) Universitat de Barcelona (UB) Barcelona Spain; 3 Àrea de sistemes d’informació Servei Català de la Salut Barcelona Spain; 4 Eurecat Technology Center of Catalonia Barcelona Spain

**Keywords:** home hospitalization, health risk assessment, predictive modeling, chronic care, integrated care, modeling, hospitalization, health risk, prediction, mortality, clinical decision support

## Abstract

**Background:**

Home hospitalization is widely accepted as a cost-effective alternative to conventional hospitalization for selected patients. A recent analysis of the home hospitalization and early discharge (HH/ED) program at Hospital Clínic de Barcelona over a 10-year period demonstrated high levels of acceptance by patients and professionals, as well as health value-based generation at the provider and health-system levels. However, health risk assessment was identified as an unmet need with the potential to enhance clinical decision making.

**Objective:**

The objective of this study is to generate and assess predictive models of mortality and in-hospital admission at entry and at HH/ED discharge.

**Methods:**

Predictive modeling of mortality and in-hospital admission was done in 2 different scenarios: at entry into the HH/ED program and at discharge, from January 2009 to December 2015. Multisource predictive variables, including standard clinical data, patients’ functional features, and population health risk assessment, were considered.

**Results:**

We studied 1925 HH/ED patients by applying a random forest classifier, as it showed the best performance. Average results of the area under the receiver operating characteristic curve (AUROC; sensitivity/specificity) for the prediction of mortality were 0.88 (0.81/0.76) and 0.89 (0.81/0.81) at entry and at home hospitalization discharge, respectively; the AUROC (sensitivity/specificity) values for in-hospital admission were 0.71 (0.67/0.64) and 0.70 (0.71/0.61) at entry and at home hospitalization discharge, respectively.

**Conclusions:**

The results showed potential for feeding clinical decision support systems aimed at supporting health professionals for inclusion of candidates into the HH/ED program, and have the capacity to guide transitions toward community-based care at HH discharge.

## Introduction

### Home Hospitalization and Early Discharge at the Hospital Clinic of Barcelona

Home hospitalization (HH)/early discharge (ED) programs [1-6] show substantial site heterogeneities in terms of service workflows and organizational aspects. However, overall, they have demonstrated maturity and health care value generation [7] such that it is well accepted that HH/ED constitutes an effective alternative to inpatient care for a select group of patients requiring hospital admission.

The characteristics of the deployment and adoption of the HH/ED program at Hospital Clinic of Barcelona (HCB) were recently described in a report [8]. In this report, HH/ED is defined as a service providing acute, home-based, short-term, complex interventions aimed at substituting conventional hospitalization fully with HH [7,9] or partially with ED [10]. The service at HCB is delivered by trained hospital personnel, and it is provided for a period of time that is not longer than the expected length of hospital stay for the patients’ diagnostic related groups involved [11]. The Hospital retains the entire clinical, fiscal, and legal responsibilities. Virtual beds are used to support the required administrative and clinical processes. The report concluded that HH/ED for acute medical and surgical patients in a real-world setting was safe, generated healthcare efficiencies, and was well accepted by 98% of patients and professionals [8]. Moreover, the study stressed the potential of HH/ED to strengthen care coordination between highly specialized hospital-based care and home-based services involving different levels of complexity [8].

Currently, the HH/ED program at HCB is a mainstream, mature service that is offered 24 hours a day, 7 days a week, all year round, with 48 virtual beds available per day. It is the first choice for eligible patients requiring hospital admission when attended in the Emergency Department, and it serves the entire Health district of Barcelona Eixample-Esquerra, which has 540,000 inhabitants.

It is well accepted that the key health outcomes that define the success of hospitalization at home [8] are mortality and unplanned emergency room consultations that lead to in-hospital admissions, either during the home hospitalization episode or during the 30-day period after discharge. This study relies on the assumption that multisource predictive modeling facilitating clinical decision support at 2 key time points—(1) at entry, and (2) at HH/ED discharge—could be useful to enhance service outcomes. Risk assessment at entry may contribute to reducing undesirable events during the episode of HH/ED, whereas the assessment of unexpected events after discharge will likely contribute to improving transitional care [12,13] and better definition of personalized care pathways within a care continuum scenario [14].

### The Use of Multisource Predictive Modeling for Enhanced Risk Assessment

This study was designed to elaborate and assess the potential of a machine learning approach to the prediction of mortality and hospital admission at entry and at discharge from HH/ED. A key specificity of the study is the use of various data sources to estimate the 2 outcomes, mortality and hospital re-admission, as conventional inpatient care. In addition to classical clinical and biological information obtained from electronic medical records (EMR), we have also considered the inclusion of Catalan population–health risk assessment scoring, known as Adjusted Morbidity Groups (GMA) [15,16], and purposely collected data on patients’ performance and frailty.

The GMA is an open, publicly owned algorithm that does not rely on expert-based fixed coefficients. Such characteristics provide a high degree of flexibility for multisource predictive modeling and good potential for transferability to other sites, as demonstrated through its validation and current use in 13 of the 17 health regions in Spain, encompassing approximately 38,000,000 citizens [15]. It is fully operational since 2015 for health policy purposes and for clinicians in primary care workstations, providing yearly updated risk stratification with a population health orientation. It takes into account multimorbidity and complexity, that is, impact on health care, using data across health care tiers stored in the Catalan Health Surveillance System.

The approach adopted in this study was based on the hypothesis that the application of holistic strategies for subject-specific risk prediction and stratification, which consider multisource covariates influencing patient health, could increase predictive accuracy and facilitate clinical decision-making based on sound estimates of individual prognosis [17]. Developed predictive models were evaluated on a real-world database, which included all cases admitted to HH/ED at HCB from January 2009 to December 2015.

## Methods

### Dataset

Retrospective data from 1936 patients admitted to the HH/ED program at HCB from January 2009 to December 2015 (Table 1S in Multimedia Appendix 1) were considered in the analyses carried out to elaborate the predictive modeling of mortality and hospital re-admission at 2 time points: (1) at entry into HH/ED, and (2) at discharge from the HH/ED program. HH/ED at HCB is run as a transversal program, under the responsibility of the medical and nurse directors of the Hospital, serving the different clinical specialties. Patients included in the HH/ED show a broad spectrum of primary diagnoses, as displayed in Table 1S in Multimedia Appendix 1.

The potential covariates considered for predictive modeling purposes (Table 2S in Multimedia Appendix 1) encompassed 3 dimensions: (1) standard clinical and biological information obtained from EMRs; (2) patients’ functional performance and frailty data, specifically collected to characterize these patients; and (3) GMA scoring indicating multimorbidity, complexity, and patients’ allocation into the population–health risk stratification pyramid.

### Ethical Approval

The Ethical Committee for Human Research at HCB approved the study, and all participants signed an informed consent prior to any procedure. The program was registered at ClinicalTrials.gov: NCT03130283.

### Predictive Analytics Workflow

**Figure 1:**
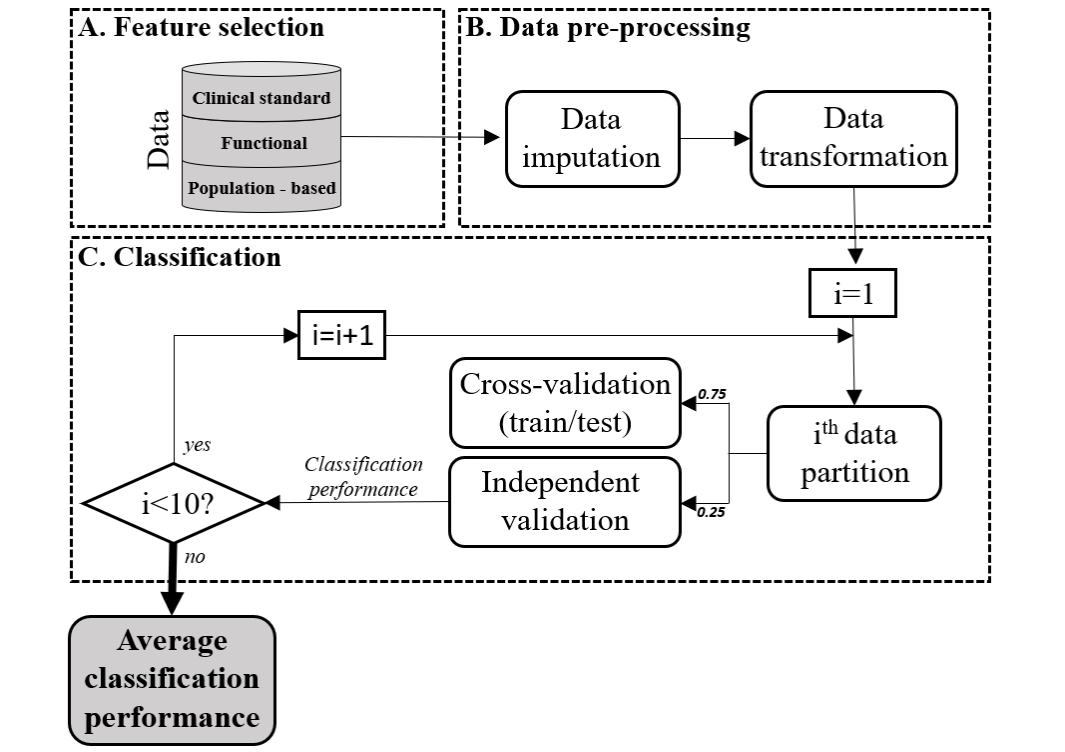
Predictive analytics workflow, composed of 3 main steps: (A) feature selection, (B) data preprocessing, and (C) classification.

Figure 1 illustrates the global methodology proposed to identify patients at risk of re-admission or death after HH discharge; the elaboration of predictive modeling followed 3 successive steps: (1) feature selection, (2) data preprocessing, and (3) classification.

#### Feature Selection

Feature selection refers to different processes involving data cleaning, selection of variables to be considered for predictive modelling, as well as selection of the final set of patients included in the analyses.

#### Data Preprocessing

In order to handle the impact of missing values, a robust method was designed for mixed-type data imputation. To this end, the missForest algorithm was applied to the whole dataset [18]. Moreover, we applied a rediscretization of some categorical variables to avoid under-represented categories.

#### Classification

Different strategies were considered for the elaboration of predictive models in this study. Specifically, 3 of them were explored in detail (Multimedia Appendix 1); that is, logistic regression and 2 machine learning approaches: a decision tree and random forest classifiers.

For model training, the dataset was 10-times divided in (1) a training subset, taking 75% of randomly selected cases, and (2) a validation subset with the remaining 25% of cases. For each data partition, the model was trained using 4-fold cross-validation on the training subset. As successful cases (ie, survivors not requiring hospital admission) were far superior in number, the effect of class imbalance was reduced by applying a random stratified-sampling strategy [19].

Model performance was assessed by computing the area under the receiver operating characteristic curve (AUROC), sensitivity, specificity, and score metrics in the validation subset. Score is a measure of prediction accuracy and is defined as the weighted harmonic mean of the sensitivity and specificity of the model. The final performance of the models was assessed as the average performance of all independent validations.

As indicated above, the methodology was applied to predict 2 types of events: (1) mortality, and (2) in-hospital admission until 30-days after HH/ED discharge. Risk assessment was conducted in 2 different scenarios: (1) at entry into the HH/ED program, and (2) at discharge. Accordingly, the analyses led to 4 different risk models (RM): (1) RM1 accounts for predicting the need for conventional hospitalization at entry into the HH/ED program; (2) RM2 predicts mortality during the study period assessed at entry; (3) RM3 refers to predictive modeling of conventional hospital admissions assessed at HH/ED discharge; and (4) RM4 predicts mortality during the study period assessed at HH/ED discharge. The risk of mortality and re-admission during HH at entry was not assessed due to the scarcity of unsuccessful cases during HH/ED.

## Results

### Study Population

All 1936 patients admitted to the HH/ED program at HCB during the study period were included in the research. However, the analyses conducted in the study were based on 1925 cases; 4 cases were discarded for having unrecoverable wrong data and 7 for having missing mandatory data. The mean age of the study group was 70.85 (SD 14.88) years; 1201 (62.4%) were men and 724 (37.6%) were women. The list of main diagnoses is depicted in Table 1S in Multimedia Appendix 1. Up to 64 variables, grouped into the 3 categories indicated above, were considered in the analyses (Table 2S in Multimedia Appendix 1).

To characterize different subpopulations of risk, patients were classified as undergoing successful and unsuccessful home hospitalization stays based on their re-admission and mortality during the study period and 30 days after hospital discharge (Tables 1-2). Of the 1925 patients admitted to the HH/ED program, 3 (0.2%) patients died and 96 (5.0%) cases were eventually readmitted to the hospital due to complications of heterogeneous origin during HH/ED. Of the remaining 1922 patients, within 30 days after HH/ED discharge, 37 (1.9%) patients died and 210 (10.9%) cases were identified as falling into the unsuccessful groups when analyzing re-admission risk. Tables 1 and 2 summarize the baseline characteristics of both study groups, according to mortality and re-admission, respectively.

Mortality was higher in elderly (*P*<.001) and comorbid patients, GMA (*P*=.02), and the Charlson Comorbidity Index (*P*=.001), especially in those with cardiovascular (*P*<.001) and oncologic disorders (*P=*.019). Mortality was lower in postoperative patients (*P*<.001) and in those with respiratory diseases (*P=*.005). Interestingly, in-hospital re-admission was only slightly associated with higher age (*P=*.003) and a major complexity of comorbid conditions, GMA (*P*<.001), and the Charlson Comorbidity Index (*P*<.001), without well-defined associations with the characteristics of the main diagnosis. 

**Table 1:** Clinical characteristics of successful and unsuccessful home hospitalization (HH) cases (n=1925) based on mortality.

Patient characteristics		Successful cases (n=1885)	Unsuccessful cases during HH (n=3)	Unsuccessful cases 30 days after HH discharge (n=37)	*P* value^a^
Age, mean (SD)		70.7 (14.9)	89.3 (15.1)	77.9 (10.6)	<.001
**Sex,** **n (%)**					
	Male	1181 (62.7)	1 (33.3)	19 (51.3)	.145
	Female	704 (37.3)	2 (66.6)	18 (48.7)	.145
GMA, mean (SD)		21.3 (13.5)	21.4 (3.1)	27.0 (14.2)	.020
Charlson Comorbidity Index, mean (SD)		4.3 (2.8)	5.7 (4.9)	5.8 (2.7)	.001
**Diagnostic group,** **n (%)**					
	Cardiology	202 (10.7)	1 (33.3)	16 (43.2)	<.001
	Respiratory	583 (30.9)	0 (0.0)	5 (13.6)	.005
	Oncology	145 (7.7)	0 (0.0)	8 (21.6)	.019
	Surgery	375 (19.9)	0 (0.0)	0 (0.0)	<.001
	Other medical acute conditions	580 (30.8)	2 (66.7)	8 (21.6)	.440

^a^*P* values were calculated comparing successful and unsuccessful groups during the full period.

**Table 2:** Clinical characteristics of successful and unsuccessful home hospitalization (HH) cases (n=1925) based on re-admission.

Patient characteristics			Successful cases (n=1638)	Unsuccessful cases during HH (n=96)	Unsuccessful cases 30 days after HH discharge (n=210)	*P* value^a^
Age, mean (SD)			70.5 (15.2)	72.9 (14.8)	73.2 (11.9)	.003
**Sex,** **n (%)**						
	Male		1007 (61.6)	63 (65.6)	142 (67.6)	.056
	Female		631 (38.4)	33 (34.4)	68 (32.4)	.056
GMA, mean (SD)			20.3 (13.1)	26.8 (15.0)	28.7 (14.7)	<.001
Charlson Comorbidity Index, mean (SD)			4.1 (2.8)	5.3 (2.6)	5.6 (2.6)	<.001
**Diagnostic group,** **n (%)**						
	Cardiology		162 (9.9)	24 (25.0)	38 (18.1)	.068
	Respiratory		507 (30.9)	24 (25.0)	62 (29.5)	.722
	Oncology		113 (6.9)	8 (8.3)	32 (15.2)	.123
	Surgery		340 (20.8)	14 (14.6)	23 (11.0)	.136
	Other medical acute conditions		516 (31.5)	26 (27.1)	55 (26.2)	.450

^a^*P* values were calculated comparing successful and unsuccessful groups during the full period.

### Predictive Modeling

Different modeling approaches were considered for this purpose, including logistic regression, decision trees, and random forests. The averaged AUROC of each modeling approach that was considered is presented in Table 3.

**Figure 2:**
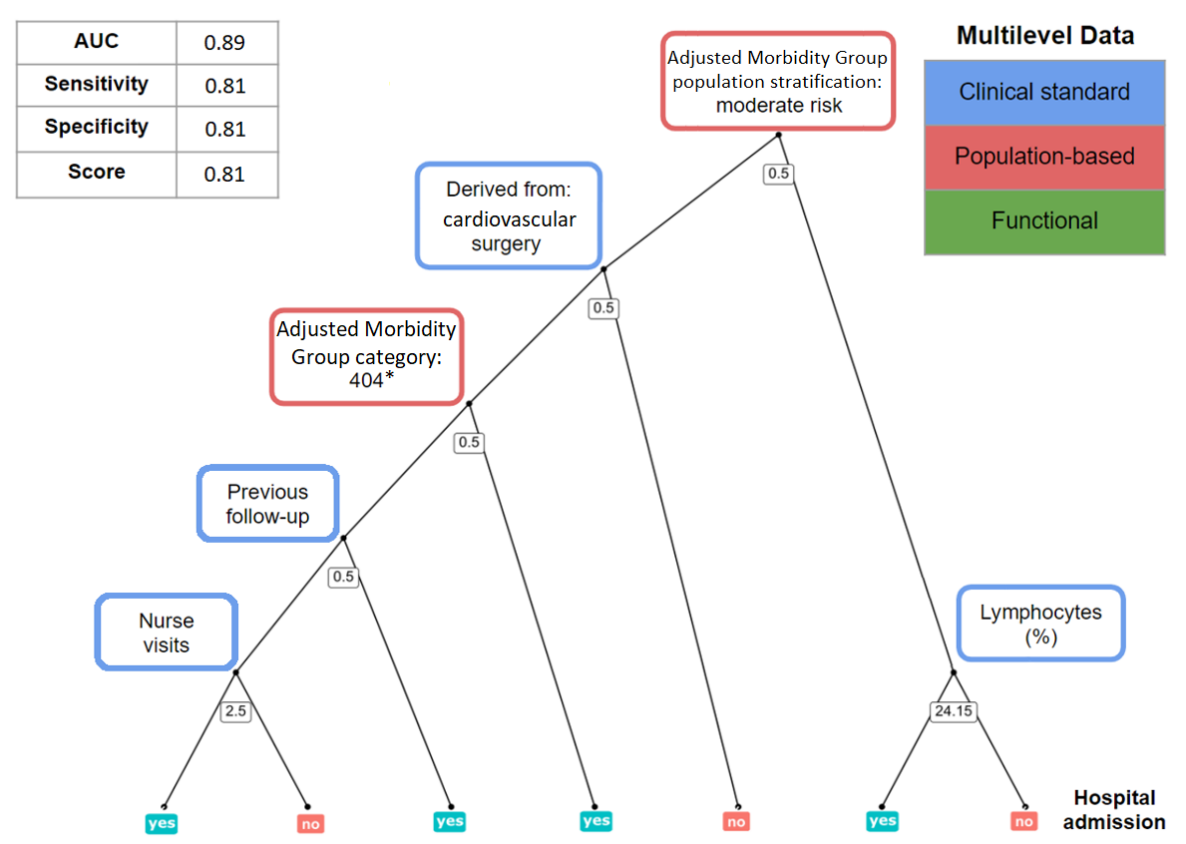
Overview of the predictive modeling strategy taking, as an example, prediction of re-admission at home hospitalization discharge. Upper-left table: metrics used for model performance assessment; AUC: area under the receiver operating characteristic curve. Center figure: representation of 1 decision tree using a random subset of features; on the nodes, threshold values for each variable determine the path from the root to the leaves (0.5 for Boolean variables), moving toward the left when the decision rule is meet; on a random forest model, final predictions are averaged over multiple decision trees. Upper-right table: 3 categories of data that are included in the models. *GMA category 404; 40: patient with active neoplasms; 4: high complexity conditions (percentile between 0.85 and 0.95).

Among the different modeling strategies developed, random forest classifier (Figure 2) showed the best performance averaged over the 4 risk scenarios.

Table 4 summarizes the performance of the 4 predictive models proposed in the study for in-hospital admission (RM1 and RM3) and for mortality (RM2 and RM4); Multimedia Appendix 2 depicts the relative weight, expressed as the mean decrease in accuracy (MDA) [20], of the 10 most relevant variables for each of the 4 predictive models.

**Table 3:** Area under the receiver operating characteristic curve (AUROC; sensitivity/specificity) performance of the modeling strategies explored.

Model	Mean AUROC (sensitivity/ specificity)	RM1 AUROC (sensitivity/ specificity)	RM2 AUROC (sensitivity/ specificity)	RM3 AUROC (sensitivity/ specificity)	RM4 AUROC (sensitivity/ specificity)
Logistic regression	0.58 (0.54/0.57)	0.65 (0.68/0.58)	0.54 (0.50/0.59)	0.59 (0.61/0.52)	0.54 (0.38/0.58)
Decision tree	0.59 (0.81/0.47)	0.62 (0.82/0.43)	0.64 (0.88/0.51)	0.57 (0.64/0.42)	0.64 (0.88/0.52)
Random forest	0.80 (0.75/0.71)	0.71 (0.67/0.64)	0.88 (0.81/0.76)	0.70 (0.71/0.61)	0.89 (0.81/0.81)

**Table 4:** Average results of the performance of the 4 home-hospitalization/early discharge (HH/ED) predictive risk models (RM).

Model	AUROC^a^, mean (SD)	Sensitivity, mean (SD)	Specificity, mean (SD)	Score, mean (SD)
Readmission risk at HH/ED admission (RM1)	0.71 (0.03)	0.67 (0.06)	0.64 (0.05)	0.66 (0.03)
Readmission risk at HH/ED discharge (RM3)	0.70 (0.02)	0.71 (0.06)	0.61 (0.05)	0.66 (0.03)
Mortality risk at HH/ED admission (RM2)	0.88 (0.04)	0.81 (0.09)	0.76 (0.04)	0.78 (0.06)
Mortality risk at HH/ED discharge (RM4)	0.89 (0.04)	0.81 (0.12)	0.81 (0.05)	0.81 (0.06)

^a^AUROC: area under the receiver operating characteristic curve.

For risk of in-hospital admissions (Multimedia Appendix 2, panels A and C), multimorbidity (expressed as GMA scoring) showed the highest predictive impact, followed by red cell distribution width (RDW). Other top predictors were polypharmacy, body mass index (BMI), a few biological variables (blood cells characteristics and glucose), and physical and mental status.

For risk of mortality (Multimedia Appendix 2, panels B and D), RDW and physical status at entry (assessed using the SF-36 questionnaire [21]) showed the highest impact in the models.

Notably, enriching the model with information acquired during HH/ED (Multimedia Appendix 2, panels C and D), several variables gained importance, such as hospital admissions during HH/ED, length of current hospitalization period, and nursing home visits.

## Discussion

### Principal Findings

The current research has developed and internally validated 4 machine learning algorithms predicting the risk of in-hospital admission and mortality for patients undergoing home-based hospitalization until 30-days after discharge from the service at HCB, from 2009 to 2015. Predictions of the 2 undesirable events were performed at 2 specific time points: at entry and at discharge from home-based hospitalization.

The study design was formulated and adopted under the hypothesis that robust predictions could be useful for clinical decision making: (1) to decide patients’ admission into the HH/ED service (RM1 and RM2); and (2) to personalize care paths for transitional care, as well as for enhanced vertical integration between specialized care and community-based services, both at patients’ discharge from HH.

A unique aspect of this research is that predictors considered in the analyses encompass 3 different categories of variables (Table 2S in Multimedia Appendix 1): (1) clinical data and biological information [22-24] extracted from patients’ electronic medical records; (2) additional variables often not considered in the clinical records specifically collected in the research protocol to reflect patients’ functional capacities and health care resources; and (3) information from GMA, the population-based, health-risk assessment tool developed and implemented in Catalonia (ES) [15,16,25].

We understand that the multisource approach adopted in this research was the most appropriate to elaborate predictive modeling in a highly heterogeneous group of patients undergoing HH/ED, in terms of clinical diagnosis and frailty status [8]. The results depicted in Table 4, in terms of AUROC and score values, indicate the reasonably good performance of the predictive models as compared to recent studies on similar scenarios [26], demonstrating the feasibility of the proposed approach and leveraging the advantages of applying machine learning in clinical risk prediction contexts in front of more traditional approaches based on standard multiple regression analyses [27]. Moreover, Multimedia Appendix 2 (panels A-D) shows a high relative contribution of variables usually not considered to be of clinical standard or relevant biological information recorded in the EMR. Overall, our results indicate that our multisource approach significantly contributes to enhanced health risk assessment with a potentially high impact on clinical decision support.

### Limitations of the Study and Lessons Learned for Clinical Application

We have not been able to identify literature on predictive modeling specifically addressing HH/ED. It may partly be due to the heterogeneity of orientations and characteristics of the ongoing HH/ED programs among sites. This fact constitutes a limitation regarding the potential for generalization of the results of this research to other sites. However, we understand that the multisource approach undertaken in this study shows enormous potential for risk assessment regarding mortality and early re-admissions of hospitalizations in general, and may show high applicability beyond the field of HH/ED. The predictive modeling undertaken in the study should be useful for defining the characteristics of personalized care paths of transitional care after hospital discharge. As indicated above, the results can have a high impact on shaping the interactions between specialized and community-based care in patients with high risk for hospital re-admissions.

A major general limitation of machine learning approaches such as the one proposed here is the fact that they can be considered “black-box” solutions, difficult to interpret by clinicians. Our work, however, is based on random forest models that provide interpretable information regarding variable importance (Multimedia Appendix 2, panels A-D) and even model visualization, thus facilitating the understanding of their predictions. We believe that the clinical interpretation of the predictors may require different approaches; for example, variables like age and diagnosis should be individually assessed for clinical judgment, while others, like the different GMA parametrization (including the Charlson Index), should be assessed by taking the category as a whole (and likewise, abnormalities in some blood test variables). On the other hand, this study indicates that the impact of patients’ functional status on outcomes is high. However, some of the measurements included in this category are not scalable in the clinical scenario (ie, SF36). Therefore, our results clearly indicate that surrogates with higher applicability [28,29] should likely be considered for inclusion in real-life clinical settings. This could be achieved through patients’ self-tracking equipment (ie, apps) that provides information on different dimensions characterizing the functional performance of the patient, namely physical and psychological status, wellbeing, activation, etc.

It is acknowledged that the generalization of the use of new clinical scores generated from predictive modeling needs external validation on other patient cohorts or in different timeframes, and even on the development of impact studies in real-world settings [30]. Apart from being costly, such a validation process can show limitations partly due to rapidly evolving clinical environments, as is the case for HH/ED at HCB, expanded to the entire health district of Eixample-Esquerra during 2018. The new scenario implies great changes in the clinical environment, patients’ characteristics, and data sources prompting the need for designing dynamic models in the context of learning health systems (LHS) [31,32]. It is of note that within a mature digital health scenario, the multisource predictive modeling approach could be enriched with other sources of data, such as patient self-reported data and data from social care. The lack of digital maturity of the current ecosystems constitutes a limiting factor for now, but in the near future, risk assessment tools are expected to improve in terms of robustness, potential for generalization of the results, and incorporation of a dynamic predictive approach.

### Steps Toward Dynamic Learning Health Systems

There is little doubt about the high potential shown by the digital transformation of health as part of a large-scale adoption of integrated care. It is acknowledged, however, that practical applications of this vision face major limitations when it comes to accessing and mining health data stored in distributed silos of information. However, it seems clear that integrating and analyzing highly complex data would open new avenues for digital health in the clinical arena.

The integration of biomedical research information systems with in-place electronic health records in hospitals and in primary care centers having interoperability with patients’ self-tracking information would enable the development of innovative, dynamic predictive modeling approaches, opening up entirely new and fascinating scenarios for an interplay between clinical practice and biomedical research [33,34]**.** We have identified 4 main interrelated enablers of this scenario [15,17,35]: (1) cloud-based tools and services allowing secure analysis of patient-centric distributed and multi-disciplinary health-related information; (2) systems medicine approaches to generate clinical predictive modeling to feed clinical decision support systems and patient decision support systems; (3) implementation and evaluation strategies for real-world implementation and assessment of cloud-based services, and (4) governance, regulatory aspects, and service adoption throughout the health care systems; these are all key to harnessing the strengths and opportunities of LHS**.**

Combined actions involving organizational changes with the engagement of all stakeholders, selective adoption of novel biomedical and digital tools, and the achievement of financial sustainability through enhanced accountability and entrepreneurial actions should pave the way toward the transition to LHS.

### Conclusions

This study proves the potential of the proposed multisource machine-learning models for the prediction of risk of re-admissions and deaths in patients undergoing home-based hospitalization in a real-world setting. Further steps beyond this study include the development of dynamic clinical decision support systems allowing progression towards sustainable patient-centered health care services.

## Appendix

Multimedia Appendix 1 On-line supplementary material: Multisource Predictive Modelling of Health Outcomes from Home Hospitalization.

Multimedia Appendix 2 Relative importance measures based on the mean decrease in accuracy (MDA) of the 10 most relevant variables for each model. GMA: morbidity adjusted group; BMI: body mass index; RDW: red cell distribution width; HH: home hospitalization.

## Abbreviations

AUROC: area under the receiver operating characteristic curve
ED: early discharge
EMR: electronic medical records
GMA: Adjusted Morbidity Groups
HCB: Hospital Clínic de Barcelona
HH: Home Hospitalization
LHS: Learning Health Systems
MDA: mean decrease in accuracy
RDW: red cell distribution width
RM: risk model

## References

[1] Leff B, Burton L, Mader SL, Naughton B, Burl J, Inouye SK, Greenough WB, Guido S, Langston C, Frick K, Steinwachs D, Burton JR (2005). Hospital at Home: Feasibility and Outcomes of a Program To Provide Hospital-Level Care at Home for Acutely Ill Older Patients. Ann Intern Med.

[2] Caplan GA, Sulaiman NS, Mangin DA, Aimonino Ricauda N, Wilson AD, Barclay L (2012). A meta‐analysis of “hospital in the home”. Medical Journal of Australia.

[3] Cryer L, Shannon SB, Van Amsterdam M, Leff B (2012). Costs for 'hospital at home' patients were 19 percent lower, with equal or better outcomes compared to similar inpatients. Health Affairs (Millwood).

[4] Jacobs JM, Hammerman-Rozenberg R, Stessman J (2006). Home Hospitalization: 15 Years of Experience. Annals of Internal Medicine.

[5] Jacobs JM, Cohen A, Rozengarten O, Meiller L, Azoulay D, Hammerman-Rozenberg R, Stessman J (2007). Closure of a home hospital program: Impact on hospitalization rates. Archives of Gerontology and Geriatrics.

[6] Montalto M (2010). The 500‐bed hospital that isn't there: the Victorian Department of Health review of the Hospital in the Home program. Medical Journal of Australia.

[7] Shepperd S, Iliffe S, Doll HA, Clarke MJ, Kalra L, Wilson AD, Gonçalves-Bradley DC (2016). Admission avoidance hospital at home. Cochrane Database Systematic Reviews.

[8] Hernández C, Aibar J, Seijas N, Puig I, Alonso A, Garcia-Aymerich J, Roca J (2018). Implementation of Home Hospitalization and Early Discharge as an Integrated Care Service: A Ten Years Pragmatic Assessment. International Journal of Integrated Care.

[9] Leff B, Montalto M (2004). Home hospital-toward a tighter definition. Journal of the American Geriatrics Society.

[10] Gonçalves-Bradley D, Iliffe S, Doll H, Broad J, Gladman J, Langhorne P, Richards SH, Shepperd S (2017). Early discharge hospital at home. Cochrane Database of Systematic Reviews.

[11] Fetter R, Shin Y, Freeman J, Averill R, Thompson J (1980). Case mix definition by diagnosis-related groups. Med Care.

[12] Yang H, Dervin G, Madden S, Beaulé PE, Gagné S, Crossan ML, Fayad A, Wheeler K, Afagh M, Zhang T, Taljaard M (2018). Postoperative Home Monitoring After Joint Replacement: Feasibility Study. JMIR Perioperative Medicine.

[13] Lopez KN, O'Connor M, King J, Alexander J, Challman M, Lovick DK, Goodly N, Smith A, Fawcett E, Mulligan C, Thompson D, Fordis M (2018). Improving Transitions of Care for Young Adults With Congenital Heart Disease: Mobile App Development Using Formative Research. JMIR Formative Research.

[14] Taylor A, Broadbent M, Wallis M, Marsden E (2019). The predictive validity of the interRAI ED screener for predicting re-presentation within 28 days for older adults at a regional hospital emergency department. Australasian Emergency Care.

[15] Dueñas-Espín I, Vela E, Pauws S, Bescos C, Cano I, Cleries M, Contel JC, de MKE, Garcia-Aymerich J, Gomez-Cabrero D, Kaye R, Lahr MMH, Lluch-Ariet M, Moharra M, Monterde D, Mora J, Nalin M, Pavlickova A, Piera J, Ponce S, Santaeugenia S, Schonenberg H, Störk S, Tegner J, Velickovski F, Westerteicher C, Roca J (2016). Proposals for enhanced health risk assessment and stratification in an integrated care scenario. BMJ Open.

[16] Monterde D, Vela E, Clèries M, Garcia-Eroles L, Roca J, Pérez-Sust P (2020). Multimorbidity as a predictor of health service utilization in primary care: a registry-based study of the Catalan population. BMC Family Practice.

[17] Cano I, Tenyi A, Vela E, Miralles F, Roca J (2017). Perspectives on Big Data applications of health information. Current Opinion in Systems Biology.

[18] Stekhoven DJ, Buhlmann P (2011). MissForest--non-parametric missing value imputation for mixed-type data. Bioinformatics.

[19] Calvo M, Cano I, Hernandez C (2019). Class Imbalance Impact on the Prediction of Complications during Home Hospitalization: A Comparative Study.

[20] Breiman L (2001). Random forests. Machine Learning.

[21] Alonso J, Prieto L, Antó J, Alonso J, Antó J (1995). La versión española del SF-36 HealthSurvey (Cuestionario de salud SF36): un instrumento para la medida de los resultados clínicos. Medicina Clinica (Barcelona).

[22] Ryckwaert F, Boccara G, Frappier JM, Colson PH (2002). Incidence, risk factors, and prognosis of a moderate increase in plasma creatinine early after cardiac surgery*. Critical Care Medicine.

[23] Yamanaka T, Matsumoto S, Teramukai S, Ishiwata R, Nagai Y, Fukushima M (2007). The Baseline Ratio of Neutrophils to Lymphocytes Is Associated with Patient Prognosis in Advanced Gastric Cancer. Oncology.

[24] González-Ferrer JJ, García-Rubira JC, Balcones DV, Gil IN, Barrio RC, Fuentes-Ferrer M, Fernández-Ortiz A, Macaya C (2008). Influence of Hemoglobin Level on In-Hospital Prognosis in Patients With Acute Coronary Syndrome. Revista Española de Cardiología (English Edition).

[25] Monterde D, Vela E, Clèries M, grupo colaborativo GMA (2016). Adjusted morbidity groups: A new multiple morbidity measurement of use in Primary Care. Atención Primaria.

[26] Min X, Yu B, Wang F (2019). Predictive Modeling of the Hospital Readmission Risk from Patients’ Claims Data Using Machine Learning: A Case Study on COPD. Scintific Reports.

[27] Reilly BM, Evans AT (2006). Translating Clinical Research into Clinical Practice: Impact of Using Prediction Rules To Make Decisions. Annals of Internal Medicine.

[28] Foebel AD, Hirdes JP, Heckman GA, Kergoat M, Patten S, Marrie RA (2013). Diagnostic data for neurological conditions in interRAI assessments in home care, nursing home and mental health care settings: a validity study. BMC Health Services Ressearch.

[29] Berwick DM, Nolan TW, Whittington J (2008). The Triple Aim: Care, Health, And Cost. Health Affairs.

[30] Bleeker S, Moll H, Steyerberg E, Donders A, Derksen-Lubsen G, Grobbee D, Moons K (2003). External validation is necessary in prediction research: A clinical example. Journal of Clinical Epidemiology.

[31] Smith M, Saunders R, Stuckhardt L, McGinnis J (2013). Best Care at Lower Cost: The Path to Continuously Learning Health Care in America.

[32] Maddox TM, Albert NM, Borden WB, Curtis LH, Ferguson TB, Kao DP, Marcus GM, Peterson ED, Redberg R, Rumsfeld JS, Shah ND, Tcheng JE (2017). The Learning Healthcare System and Cardiovascular Care: A Scientific Statement From the American Heart Association. Circulation.

[33] Auffray C, Charron D, Hood L (2010). Predictive, preventive, personalized and participatory medicine: back to the future. Genome Medicine.

[34] Aarestrup FM, Albeyatti A, Armitage WJ, Auffray C, Augello L, Balling R, Benhabiles N, Bertolini G, Bjaalie JG, Black M, Blomberg N, Bogaert P, Bubak M, Claerhout B, Clarke L, De Meulder B, D’Errico G, Di Meglio A, Forgo N, Gans-Combe C, Gray AE, Gut I, Gyllenberg A, Hemmrich-Stanisak G, Hjorth L, Ioannidis Y, Jarmalaite S, Kel A, Kherif F, Korbel JO, Larue C, Laszlo M, Maas A, Magalhaes L, Manneh-Vangramberen I, Morley-Fletcher E, Ohmann C, Oksvold P, Oxtoby NP, Perseil I, Pezoulas V, Riess O, Riper H, Roca J, Rosenstiel P, Sabatier P, Sanz F, Tayeb M, Thomassen G, Van Bussel J, Van den Bulcke M, Van Oyen H (2020). Towards a European health research and innovation cloud (HRIC). Genome Medicine.

[35] Habl C, Renner AT, Bobek J, Laschkolnig A (2016). Study on Big Data in Public Health, Telemedicine and Healthcare. European Comission.

