# Supplementary material for: Health Outcomes from Home Hospitalization: Multisource Predictive Modeling
Source: J Med Internet Res. 2020 Oct 7;22(10):e21367. doi: 10.2196/21367 (PMC7578817; doi:10.2196/21367)
Supplement: Multimedia Appendix 1 [file jmir_v22i10e21367_app1.docx]

**Health Outcomes from Home Hospitalization: Multisource Predictive Modelling**

# Mireia Calvo et al.

*(On-line supplementary material)*

The current on-line supplementary material provides detailed information on the methodological approach adopted in the current study, as well as additional data complementing the description of the results showed in the main manuscript.

## *METHODS*

## *Predictive analytics workflow*


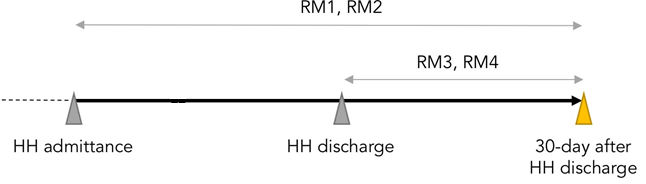
The methodology was applied in four scenarios, leading to four different models (RM1-RM4), represented in *Figure 1S.* Each model was designed for the identification of patients undergoing two types of unsuccessful events: mortality (RM2, RM4) and in-hospital admissions (RM1 and RM3) up to 30-days after home hospitalization discharge. Patients were assessed at HH admittance (RM1 and RM2 for in-hospital admissions and mortality, respectively) and at home-hospitalization discharge (RM3 and RM4 for in-hospital admissions and mortality, respectively).

*Figure 1S.* *Elaborated risk models (RM). RM1 accounts for the model predicting in-hospital admissions during the study period at entry (HH admittance); RM2 predicts mortality risk at HH admittance; RM3 and RM4 respectively refer to readmission and mortality predictions at HH discharge.*

*Feature selection*

The list of main diagnosis in the study group is described in *Table 1S,* where up to 160 ICD-9-CM [1] items were identified and classified by biological systems [2]**.** Health-risk assessment was based on a multisource solution relying on the hypothesis that subject-specific risk prediction and stratification could be significantly improved by considering multisource covariates influencing patients’ health, namely: (i) standard clinical and biological variables, (ii) patients’ functional performance; and, (iii) population-based information. To this end, up to 65 potential predictors displayed in *Table 2S* were considered in the analyses. This table also indicates the two outcome variables: mortality and in-hospital admission.

*Table 1S.* Main diagnosis in the study group.

| *System (ICD-9-CM classification)* | *Cases n (%)* |
| --- | --- |
| *Circulatory* | 514 (26.7%) |
| Cardiac Insufficiency (428; 428.0; 428.1; 429.83; 398.91) | 187 (9.71%) |
| Heart valve diseases (424; 424.0; 424.1; 394.2; 396.3; 425.11; 746.4) | 83 (4.31%) |
| Ischaemic heart disease; angina (410; 410.11; 410.41; 410.42; 410.71; 410.72; 410.91; 414; 414.0; 414.01) | 82 (4.26%) |
| Acute cor pulmonale (415.1; 415.95) | 53 (2.75%) |
| Chronic pulmonary Heart Disease (415.96; 416; 416.8) | 26 (1.35%) |
| [Atherosclerosis; of native arteries of the extremities](https://www.findacode.com/code-set.php?set=ICD9&i=1049) (440.02) | 25 (1.3%) |
| [Pulmonary embolism and infarction](https://www.findacode.com/code-set.php?set=ICD9&i=1003) (415.19; 674.84) | 17 (0.88%) |
| [Acute venous embolism and thrombosis of deep vessels of lower extremity](https://www.findacode.com/code-set.php?set=ICD9&i=7193) (453.4; 453.40;448) | 15 (0.78%) |
| Thrombophlebitis migrans (453.41) | 8 (0.42%) |
| [Iatrogenic hypotension.](https://www.findacode.com/code-set.php?set=ICD9&i=7194) (958.3) | 7 (0.36%) |
| Cardiac Arrhythmia (427.31) | 3 (0.16%) |
| Atheroembolism (445.5) | 2 (0.1%) |
| Arterial embolism and thrombosis (444; 444.22) | 2 (0.1%) |
| Unspecified hypertensive heart disease with heart failure (402.91) | 2 (0.1%) |
| [Acute and subacute endocarditis](https://www.findacode.com/code-set.php?set=ICD9&i=1008) (421) | 1 (0.05%) |
| Abdominal aneurysm without mention of rupture (441.4) | 1 (0.05%) |
| *Endocrine; nutritional and metabolic* | 8 (0.42%) |
| Diabetes with other specified manifestations. (250.8; 277.89; 276.9) | 8 (0.42%) |
| *Cancers (and sequelae)* | 7 (0.36%) |
| Cancer in any site (153.9; 188.8; 225.2; 338.3) | 7 (0.36%) |
| *Respiratory* | 882 (45.82%) |
| Chronic Obstructive Pulmonary Disease Exacerbation (491.21; 492.8; 491.22) | 345 (17.92%) |
| Pneumonia (481; 481.0; 482.2; 482.30; 482.83; 482.84; 486; 486.0) | 165 (8.57%) |
| Acute respiratory infections (466; 466.0) | 91 (4.73%) |
| Respiratory system disease (519.8; 519.9) | 82 (4.26%) |
| Bronchiectasis with acute exacerbation (494.1) | 51 (2.65%) |
| Asthma exacerbation (493.02; 493.22; 493.91; 493.92; 493.01) | 50 (2.6%) |
| Pneumonitis due to solids and liquids (507; 507.0; 502) | 30 (1.56%) |
| Flu and Influenza (487.0; 487.1; 488; 488.19; 488.81; 488.82) | 21 (1.09%) |
| Other alveolar and parietoalveolar pneumonopathy (516) | 16 (0.83%) |
| Other diseases of respiratory system (518.81; 518.89; 519; 516.31; 518.4) | 15 (0.78%) |
| Alveol pneumonopathy (516.8) | 8 (0.42%) |
| Segmental resection of lung (32.3) | 4 (0.21%) |
| Hemoptysis (786.3; 786.30; 786.39) | 3 (0.16%) |
| Tracheostomy complications (519.19) | 1 (0.05%) |
| *Digestive* | 30 (1.56%) |
| Cholangitis (576.1) | 9 (0.47%) |
| [Other specified disorders of intestine](https://www.findacode.com/code-set.php?set=ICD9&i=7215) (569.81; 51.23; 557.0; 542) | 6 (0.31%) |
| Non-infectious gastroenteritis (558.9) | 4 (0.21%) |
| Intestinal infectious diseases (003.0; 009; 566) | 4 (0.21%) |
| Liver Cirrhosis (572; 572.1; 572.2) | 3 (0.16%) |
| [Ventral hernia](https://www.findacode.com/code-set.php?set=ICD9&i=7208) (553.2) | 2 (0.1%) |
| [Complete edentulism](https://www.findacode.com/code-set.php?set=ICD9&i=1184) (425.4) | 2 (0.1%) |
| *Musculoskeletal system and connective tissues* | 8 (0.42%) |
| Other disorders of bone and cartilage (733.14; 733.19; 715.95; 730; 84.15; 996.76) | 7 (0.36%) |
| Inflammatory spondylopathy (720.9) | 1 (0.05%) |
| *Disease of the skin and subcutaneous tissue* | 40 (2.08%) |
| Other cellulitis and abscess (682; 682.2; 682.3; 682.6; 682.9; 881.11) | 40 (2.08%) |
| *Genitourinary* | 253 (13.14%) |
| Urine tract infection NOS (599; 599.0; 599.9; 112.2; 601.9) | 174 (9.04%) |
| Infection of kidney NOS (590.1; 590.10; 580.9; 614.9; 584.8; 590.2) | 58 (3.01%) |
| Acute prostatitis (601; 601.0) | 12 (0.62%) |
| Fournier gangrene (688.83) | 5 (0.26%) |
| Radical prostatectomy (60.5) | 2 (0.1%) |
| [Other orchitis, epididymitis, and epididymo-orchitis, without mention of abscess](https://www.findacode.com/code-set.php?set=ICD9&i=7224) (604.9; 604.90) | 2 (0.1%) |
| *Complications* | 88 (4.57%) |
| Supplementary classification of factors influencing health status & contact with health services (V45.89; V49.7; V49.72; V67.09) | 43 (2.23%) |
| Persons encountering health services for specific procedures and aftercare (V55.3; V58.49; V58.73; V58.74) | 28 (1.45%) |
| Complications of surgical and Medical Care. Not Elsewhere classified (997.49; 997.5; 997.62; 998.59; 998.6; 999; 999.31) | 17 (0.88%) |
| *Other* | 95 (4.94%) |
| Diseases of the blood and blood-forming organs (288; 288.03) | 48 (2.49%) |
| Symptoms, signs, and ill-defined conditions (780.6; 780.60; 787.91) | 42 (2.18%) |
| Infectious and parasitic diseases (31.0; 38.0; 38.49; 38.8; 86.83) | 5 (0.26%) |

*Table 2S.* Variables description and classification.

| *Variable* | *Description* |
| --- | --- |
| *Standard clinical and biological variables* | |
| *Sex* | Patient’s sex (categorical, 2 levels) |
| *Age* | Patient’s age (numerical) |
| *entr_g* | Entrance gate to hospital (categorical, 3 levels) |
| *Service* | Hospital service of origin (categorical, 13 levels) |
| *days_hospital* | Number of days in hospital (numerical) |
| *days_HH* | Number of days hospitalized at home (numerical) |
| *days_tot* | Total hospitalization days (numerical) |
| *year_cat* | Year of admission (categorical, 7 levels) |
| *diag_cat* | Diagnostic group (categorical, 5 levels) |
| *N_diag* | Number of secondary diagnoses (numerical) |
| *Smoking* | Smoking habit (categorical, 3 levels) |
| *BMI* | Body mass index (numerical) |
| *equip_pre* | Previous respiratory therapy at home (categorical, 10 levels) |
| *ox_pre* | Previous oxygen therapy at home (categorical, 2 levels) |
| *vent_pre* | Previous non-invasive ventilation at home (categorical, 3 levels) |
| *Ntecn_home* | Number of self-management techniques at home (numerical) |
| *equip_HH* | Medical equipment setting during home hospitalization (categorical, 5 levels) |
| *equip_dis* | Medical equipment setting at discharge (categorical, 6 levels) |
| *diff_med* | Difficulty in taking treatment (categorical, 2 levels) |
| *pills_day* | Number of pills per day (numerical) |
| *inj_day* | Number of injections per day (numerical) |
| *inh_day* | Number of inhalations therapy per day (numerical) |
| *atb_ev* | Intravenous antibiotic during home hospitalization (categorical, 4 levels) |
| *furo_ev* | Intravenous furosemide (categorical, 2 levels) |
| *cort_ev* | Intravenous corticosteroids (categorical, 2 levels) |
| *treat_ev* | Other intravenous treatment (categorical, 2 levels) |
| *Heparin* | Subcutaneous heparin (categorical, 2 levels) |
| *Cures* | Requires wound during home hospitalization (categorical, 2 levels) |
| *Spirometry* | Forced spirometry performed during home hospitalization (categorical, 2 levels) |
| *Gasometry* | Arterial Blood Gases performed during home hospitalization (categorical, 2 levels) |
| *name_patho* | Patient knows the name of his/her chronic disease (categorical, 2 levels) |
| *alar_sig* | Patient knows the warning signs of his/her chronic disease (categorical, 2 levels) |
| *emerg_pre* | Previous visits to emergency room, in the last 12 months (numerical) |
| *adm_pre* | Previous hospital admissions, in the last 12 months (categorical, 2 levels) |
| *emerg_HH* | Visits to emergency room during home hospitalization (categorical, 2 levels) |
| *adm_HH* | In-hospital admissions during home hospitalization (categorical, 2 levels) |
| *vis_nurs* | Number of nursing home visits during home hospitalization (numerical) |
| *vis_doc* | Number of Physician home visits during home hospitalization (numerical) |
| *vis_hosp* | Number of hospital visits during home hospitalization (numerical) |
| *Call* | Number of phone calls from professionals during home hospitalization (numerical) |
| *followup_pre* | Follow-up, previous to home hospitalization (categorical, 9 levels) |
| *followup_dis* | Follow-up, after home hospitalization discharge (categorical, 9 levels) |
| *followup_ca* | Follow-up category (categorical, 4 levels) |
| *ABS* | Health Basic Area (categorical, 21 levels) |
| *AGA* | Assistance Management Area (categorical, 4 levels) |
| *Ref_hospital* | Reference hospital (categorical, 4 levels) |
| *Provider* | Service provider (categorical, 5 levels) |
| *disease_group* | Disease group, based on ICD-9-CM classification (categorical, 10 levels) |
| *Leu* | Leukocyte count (numerical) |
| *Lym* | Percentage of lymphocytes (numerical) |
| *Hb* | Hemoglobin concentration (numerical) |
| *RDW* | Red cell distribution width (numerical) |
| *Glu* | Glucose (numerical) |
| *Cr* | Creatinine (numerical) |
| *Na* | Sodium (numerical) |
| *K* | Potassium (numerical) |
| *Charlson* | Charlson index (numerical) |
| *Patients’ functional characteristics* | |
| *Walk* | Patient walks regularly (categorical, 2 levels) |
| *Barthel* | Barthel index (numerical) |
| *p_state* | Physical state, based on SF-36 questionnaire (numerical) |
| *m_state* | Mental state, based on SF-36 questionnaire (numerical) |
| *Population-based data* | |
| *GMA_cat* | GMA category, based on the chronicity, complexity and number of physiological systems affected by underlying diseases (categorical, 21 levels) |
| *GMA* | Patient’s individual risk (percentile for GMA risk pyramid) |
| *Pstrat* | Risk category, based on GMA grading for the general population (categorical, 4 levels) |
| *Cstrat* | Risk category, based on GMA grading for this particular cohort (categorical, 4 levels) |
| *Outcomes* | |
| *readm_HH* | In-hospital admissions during home hospitalization (categorical, 2 levels) |
| *mort_HH* | Mortality during home hospitalization (categorical, 2 levels) |
| *readm_30* | Readmission 30 days after home hospitalization discharge (categorical, 2 levels) |
| *mort_30* | Mortality 30 days after home hospitalization discharge (categorical, 2 levels) |

*Data pre-processing*


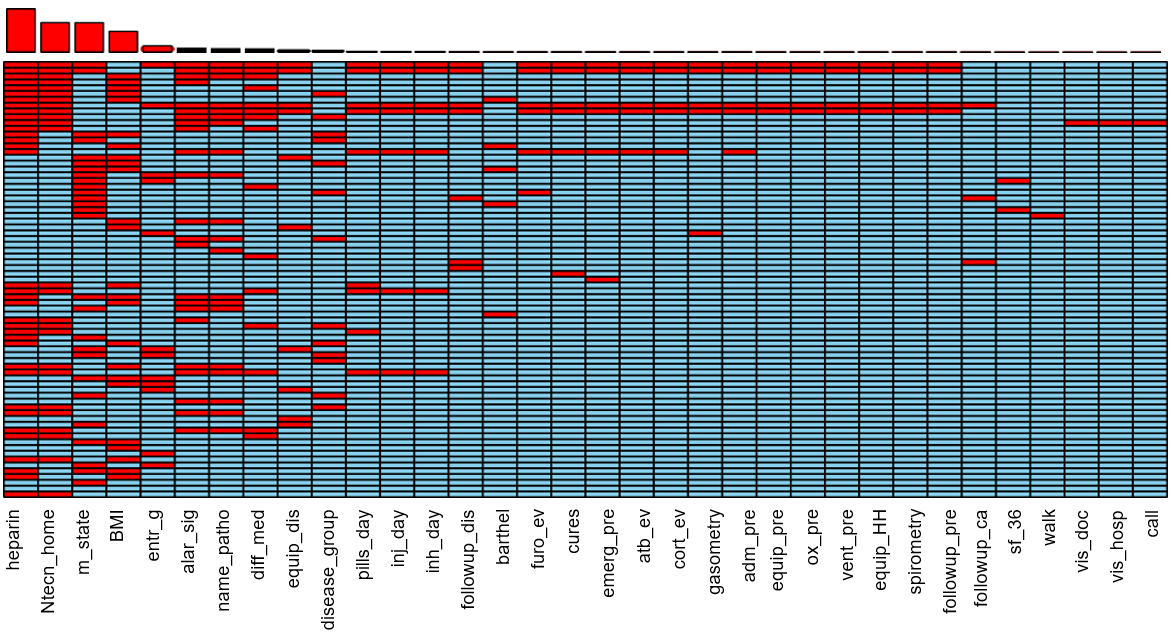
Among the 65 selected features, 34 variables, represented in *Figure 2S*, contained some missing data. Those features presenting more missings were *heparin* (45.5%), the number of applied techniques at home or *Ntecn home* (31.1%), the physical and mental state or *p_state and m_state* (31.0%) and the Body Mass Index or *BMI* (21.9%), while the other remaining thirty variables contained less than 7% of missing values. In order to reduce the impact of these missing data, a recently proposed method for data imputation, named missForest [3], was applied. Based on random forest algorithms, this non-parametric technique is capable of robustly predicting mixed-type missing values.

*Figure 2S. Patterns of missing values per variable ordered by frequency. Red squares refer to missing values. On the top, a frequency chart of missing values per variable*.

On the other hand, some categorical variables presented a great number of categories, some of them with just a few samples. In order to avoid this under-representation, we applied a re-discretization phase, grouping them to maximum of 21 categories per variable. Some of these re-discretized features were the Primary Care Unit and the GMA cat, in which some original categories were merged. Moreover, since diseases responsible for HH/ED admissions were labeled based on ICD-9-CM codes, this information was translated into 10 main disease groups (disease group). More details on this translation are provided in *Table 1S.*

*Predictive modelling approaches and classification*

In the current study, predictive modelling was defined as classifying patients in successful or unsuccessful groups according to the four scenarios explained (RM1-RM4). Different modelling approaches were considered for this purpose, including logistic regression, decision trees and random forests.

Among the classifiers that were tested, random forest models provided the best performance*.* This ensemble learning method builds a forest of uncorrelated decision trees using a CART-like procedure, combined with randomized node optimization and bagging [4]. Moreover, it allows variable importance quantification using Mean Decrease in Accuracy (MDA) method. During the fitting process of a random forest, the out-of-bag error for each data point is recorded and averaged over the forest. Thus, to measure the importance of the j*th* feature after training, the values of this j*th* feature are permuted among the training data and the out-of-bag error is again computed on this perturbed dataset. The importance score for the j*th* variable is computed by averaging the difference in out-of-bag errors before and after the permutation over all trees and the score is normalized by the standard deviation of these differences. As a result, features producing larger values for this score are ranked as more important.

Each one of the three modeling strategies were assessed using the 10-time iterative process*,* including data partition, model training and independent validation. For each iteration, processed data was randomly split in two parts: training subset, selecting 75 % of the cases, and validation subset, taking the remaining 25%.

Then, model training was conducted using 4-fold cross-validation. This technique randomly splits training data in 4 blocks and uses 3 of them for training and the remaining one for testing performance. This process is applied iteratively four times, one for each combination of training and testing split, so that each of the 4-folds is used for training and also for testing. Final performance is averaged over all the results.

In order to prevent bias from class imbalance [5], a random stratified-sampling strategy for each data partition was proposed. This technique randomly selects cases from classes separately, ensuring representation of different groups is balanced in each subset of data. Since applying this strategy provided an increase of performance, this step was added in the final classification pipeline.

The independent evaluation step was based on the resulting confusion matrix, which specifies the number of true positives (*TP*), true negatives (*TN*), false positives (*FP*) and false negatives (*FN*), when comparing true and predicted labels and considering unsuccessful HH stays as positives. First, the or area under the Receiver Operating Characteristic (ROC) curve (AUC) was computed to quantify the classifier performance. Moreover, classical sensitivity (*Se* = *TP/*(*TP* + *FN*)) and specificity (*Sp* = *TN/*(*TN* + *FP*)) measures, associated with the optimal operating point in the ROC curve, were calculated to quantify the classifier capability of correctly detecting unsuccessful and successful cases, respectively. Finally, an alternative measure sometimes used as an evaluation metric for imbalanced datasets [6], defined as the harmonic mean between sensitivity and specificity and herein named after Score (*S*), was calculated. The final performance of the models was assessed as the average performance of all independent validations.

# *REFERENCES*

1. Cherkin DC, Deyo RA, Volinn E, Loeser JD. Use of the International Classification of Diseases (ICD-9-CM) to identify hospitalizations for mechanical low back problems in administrative databases. In: Spine (Phila Pa 1976); 1992. 17(7):817–25.

2. Hernandez C, Jansa M, Vidal M, Nuñez M, Bertran MJ, Garcia-Aymerich J, et al. The burden of chronic disorders on hospital admissions prompts the need for new modalities of care: A cross-sectional analysis in a tertiary hospital. In: QJM An Int J Med; 2009. 102(3):193–202.

3. Stekhoven DJ, Buhlmann P. MissForest--non-parametric missing value imputation for mixed-type data. In: Bioinformatics; 2012. 28(1):112–8.

4. Breiman L. Random forests. Mach Learn; 2001. 45(1):5–32.

5. Calvo M, Cano I, Hernandez C, Ribas V, Miralles F, Roca J, et al. Class Imbalance Impact on the Prediction of Complications during Home Hospitalization: A Comparative Study. In: Proceedings of the Annual International Conference of the IEEE Engineering in Medicine and Biology Society, EMBS. Institute of Electrical and Electronics Engineers Inc.; 2019. p. 3446–9.

6. Clifford GD, Liu C, Moody B, Springer D, Silva I, Li Q, et al. Classification of normal/abnormal heart sound recordings: The PhysioNet/Computing in Cardiology Challenge 2016. In: Computing in Cardiology; 2016. p. 609–12.
