# Supplementary material for: Health Outcomes from Home Hospitalization: Multisource Predictive Modeling
Source: J Med Internet Res. 2020 Oct 7;22(10):e21367. doi: 10.2196/21367 (PMC7578817; doi:10.2196/21367)
Supplement: Multimedia Appendix 2 [file jmir_v22i10e21367_app2.docx]

Relative importance measures based on the mean decrease in accuracy (MDA) of the 10 most relevant variables for each model. GMA: morbidity adjusted group; BMI: body mass index; RDW: red cell distribution width; HH: home hospitalization.

|  |  |  |  |  |  |  |
| --- | --- | --- | --- | --- | --- | --- |
|  | **A – Re-admission risk at admission (RM1)** | |  | **B - Mortality risk at admission (RM2)** | |  |
|  | **Variable** | **Relative variable importance (%)** |  | **Variable** | **Relative variable importance (%)** |  |
|  | GMA | 100.00 |  | RDW | 100.00 |  |
|  | RDW | 96.54 |  | Physical state | 74.37 |  |
|  | Hemoglobin concentration | 81.23 |  | Hemoglobin concentration | 73.49 |  |
|  | Physical state | 73.82 |  | BMI | 69.14 |  |
|  | Glucose | 71.27 |  | GMA | 66.57 |  |
|  | Charlson index | 67.34 |  | Barthel index | 64.90 |  |
|  | Pills per day | 66.73 |  | Lymphocytes percentage | 62.77 |  |
|  | Potassium | 63.21 |  | Pills per day | 61.09 |  |
|  | Mental state | 61.66 |  | Charlson index | 56.32 |  |
|  | BMI | 58.98 |  | Days in hospital | 55.78 |  |
|  |  |  |  |  |  |  |
|  | **C – Re-admission risk at discharge (RM3)** | |  | **D - Mortality risk at discharge (RM4)** | |  |
|  | **Variable** | **Relative variable importance (%)** |  | **Variable** | **Relative variable importance (%)** |  |
|  | GMA | 100.00 |  | Physical state | 100.00 |  |
|  | RDW | 74.21 |  | Physician home visits | 73.05 |  |
|  | Lymphocytes percentage | 71.89 |  | Hospital admission HH | 72.78 |  |
|  | Leukocyte count | 67.46 |  | Nursing home visits | 62.66 |  |
|  | Charlson index | 66.32 |  | Total hospitalization days | 57.70 |  |
|  | BMI | 65.55 |  | Lymphocytes percentage | 55.33 |  |
|  | Nursing home visits | 62.90 |  | BMI | 54.01 |  |
|  | Potassium | 62.25 |  | Hemoglobin concentration | 53.89 |  |
|  | Glucose | 60.27 |  | Creatinine | 51.73 |  |
|  | Pills per day | 59.01 |  | Sodium | 49.87 |  |
|  |  |  |  |  |  |  |
